# Dietary habits and adherence to the Mediterranean diet in a cohort of Parkinson’s disease patients in Lithuania

**DOI:** 10.3389/fnut.2026.1773331

**Published:** 2026-03-09

**Authors:** Jevgenija Guk, Rūta Kaladytė Lokominienė, Anatolij Nečiporenko, Dalius Jatužis

**Affiliations:** Clinic of Neurology and Neurosurgery, Institute of Clinical Medicine, Faculty of Medicine, Vilnius University, Vilnius, Lithuania

**Keywords:** dietary habits, Mediterranean diet, motor symptoms, non-motor symptoms, Parkinson’s disease

## Abstract

**Background:**

Parkinson’s disease (PD) is a neurodegenerative disorder characterized by motor and non-motor symptoms. A growing body of evidence shows that a healthy diet, including the Mediterranean diet (MeDi), can slow the disease progression and improve certain motor and non-motor symptoms. There is limited knowledge about the dietary habits of patients with PD in Lithuania. We aimed to investigate nutritional habits, adherence to the MeDi, related demographic and disease-related factors in Lithuanian PD patients.

**Methods:**

The case–control study was conducted at Vilnius University Hospital Santaros Klinikos from 2023 to 2025. A food frequency questionnaire (FFQ) was used to assess dietary habits and construct the MeDi adherence score. Dietary habits and adherence to the MeDi were compared between PD patients and controls. The association between MeDi adherence and the odds of PD, severity of motor and non-motor symptoms, was assessed.

**Results:**

A total of 59 patients with PD and 54 healthy controls (HC) were recruited. The MeDi score in the HC group was slightly higher than in the PD group (31.70 vs. 29.62, *p* = 0.058), and adherence was low in both groups. Respondents in the PD group consumed fewer potatoes (*p* = 0.009) and alcohol (*p* = 0.016) and more fruits (*p* = 0.012), poultry (*p* = 0.004), and olive oil (*p* = 0,024). A statistically significant reduction in PD odds emerged after adjustment for smoking, BMI, and physical activity (OR = 0.92, 95% CI 0.85–0.99, *p* = 0.042), and remained significant in the fully adjusted model including education and income (OR = 0.90, 95% CI 0.81–0.98, *p* = 0.025). Higher MeDi adherence was associated with lower odds of pain (OR = 0.86, 95% CI 0.75–0.98), urinary dysfunction (OR = 0.86, 95% CI 0.75–0.99), constipation (OR = 0.88, 95% CI 0.78–0.99), and anxiety (OR = 0.88, 95% CI 0.79–0.99).

**Conclusion:**

Patients with PD had different dietary habits compared to controls. However, adherence to the MeDi diet was low in the elderly Lithuanian population (> 65 years), including patients with PD. Higher MeDi adherence was associated with lower odds of PD and selected non-motor symptoms in multivariable analyses.

## 1 Introduction

Parkinson’s disease (PD) is a chronic neurodegenerative disorder characterized by such motor symptoms as resting tremor, bradykinesia, rigidity, postural instability, and gait disturbances (1). This is the most common movement disorder and the second most common neurodegenerative disorder after Alzheimer’s disease, with a prevalence of about 0.5–1% among individuals aged 65–69, rising to 1–3% among those aged 80 years and older (1). According to the modeling study of the Global Burden of Disease Study 2021, the number of people with PD is expected to increase between 2021 and 2050, rising from 12 million to 25 million, due to population aging and growth (2). Despite significant advances in understanding the etiopathogenesis of the disease, a lack of effective treatments for both motor and non-motor symptoms remains, and no well-defined disease-modifying strategies are currently available (3). Most drugs used to treat PD only compensate for the lack of dopamine in the nigrostriatal system and alleviate the motor symptoms, but do not prevent disease progression and usually do not affect non-motor symptoms that are independent of dopaminergic stimulation, such as sleep, mood, cognitive, autonomic, gastrointestinal, and urogenital system disorders. On the other hand, antiparkinsonian medications may cause or worsen some of the non-motor symptoms in patients with PD, and in the long term, contribute to the risk of motor fluctuations (4). It is believed that 90% of patients have an idiopathic multifactorial disease that begins due to the interaction of genetic, epigenetic, and environmental neurotoxic and neuroprotective factors (5). According to H. Braak’s hypothesis, the pathogenesis of PD begins in the gastrointestinal tract, where an unknown pathogen triggers the formation of pathological alpha-synuclein (*α*-syn) in submucosal neurons, which in turn enters the nuclei of the central nervous system through retrograde axonal transport (6). Although the exact trigger that initiates the cascade is unknown, recent attention has focused on diet and intestinal dysbiosis as potential factors in the onset and progression of PD (7). Nutrition has received increased attention, as dietary components and diet are modifiable lifestyle factors that can positively or negatively impact PD pathogenesis (8). Nutrition-related risk factors are among the primary contributors to non-communicable diseases and mortality worldwide (9). Meanwhile, a healthy diet can reduce the risk and progression of chronic non-communicable diseases, including neurodegenerative disorders (10). Based on biological hypotheses, various nutrients, foods, and food groups were examined as possible risk or protective factors. Epidemiological and experimental animal studies have identified food groups that may have a neuroprotective effect in patients with PD, or, conversely, promote neurodegeneration; however, the effectiveness of these substances has not been confirmed in clinical studies (11). Nevertheless, daily food contains many nutrients and components that interact with one another, exerting synergistic or antagonistic effects on the human body. Therefore, recently, more attention has been paid not to individual nutrients, but to studies of the entire diet in the pathogenesis of diseases. Such studies of dietary patterns consider the complexity of the diet, the synergistic effects of the nutrients that comprise it, and the interactions between them (10, 11). The biochemical effects of nutrition on PD have been recently reviewed. The components and patterns of diets may reduce and prevent the generation of *α*-synuclein by intervening in oxidative stress and inflammation, and they also play a role in regulating the development, growth, and survival of dopaminergic neurons, including the regulation of the balance of the microbiota-gut-brain axis (12). A growing body of evidence suggests that various healthy diets, including the Mediterranean diet (MeDi), have a positive impact on the risk of developing PD, disease progression, and motor and non-motor symptoms (13–29). The MeDi is rich in antioxidants and anti-inflammatory agents. Among the constituent elements of MeDi, vitamins, omega-3 polyunsaturated fatty acids (*ω*-3 PUFAs), and polyphenols have been shown to have protective properties against PD (12). Recent studies have shown that a higher MeDi score is inversely associated with the mortality rate in PD, suggesting that this dietary pattern may also improve PD (30). Although the beneficial effects of the MeDi on health-related outcomes were initially observed in countries within the Mediterranean region, they have since been confirmed in diverse non-Mediterranean countries. As a result, MeDi or MeDi-like diets have been recommended in the national guidelines of various non-Mediterranean countries, including the United States of America, Australia, and Ireland (10). The beneficial effects of the MeDi on PD have also been observed in various countries beyond the Mediterranean region, including Iran, the USA, Canada, the Netherlands, Sweden, and China (13–15, 18, 21, 22, 25, 27, 29, 31–33). Therefore, it is important to assess the MeDi adherence among patients with PD in other non-Mediterranean populations, such as Lithuanians.

Previous studies on adherence to the MeDi in Lithuania have primarily focused on adolescents and young adults, or during the COVID-19 pandemic confinement (34–36). The dietary habits and adherence to the Mediterranean diet among patients with PD in Lithuania have not yet been studied. Before considering diet as a potential therapeutic strategy, it is crucial to define and quantify current adherence to develop effective intervention strategies for improvement. Our study aimed to assess the dietary habits, specifically focusing on MeDi, among patients with PD in our clinic and their associations with motor and non-motor symptoms.

## 2 Materials and methods

### 2.1 Participants

This case–control observational study was conducted in the Parkinson’s disease center of Vilnius University Hospital Santaros klinikos from January 2023 to January 2025.

A total of 59 patients with PD and 54 healthy controls (HC) were enrolled. We consecutively included patients with idiopathic PD, diagnosed according to UK Parkinson’s Disease Society Brain Bank Diagnostic criteria, older than 50 years, Hoehn-Yahr stage I-IV, on stable antiparkinsonian medication dose for 3 months, able to speak and understand the Lithuanian language, and willing to participate in the study (37). Participants were excluded from the study if they had: atypical Parkinsonism and other Parkinsonian disorders, other central nervous system disorders, other somatic disorders that can influence dietary habits (food intolerance, diabetes mellitus, stomach ulcer, inflammatory bowel disease, irritable bowel syndrome), psychiatric conditions (psychosis, substance abuse, significant depression), individuals with moderate and severe cognitive impairment, defined as a score of ≤ 24 points on the Mini-mental State Examination (MMSE). The study included only PD patients diagnosed ≥ 2 years prior to the dietary assessment, to ensure the dietary exposure evaluated did not precede the onset of the disease.

**Figure 1:**
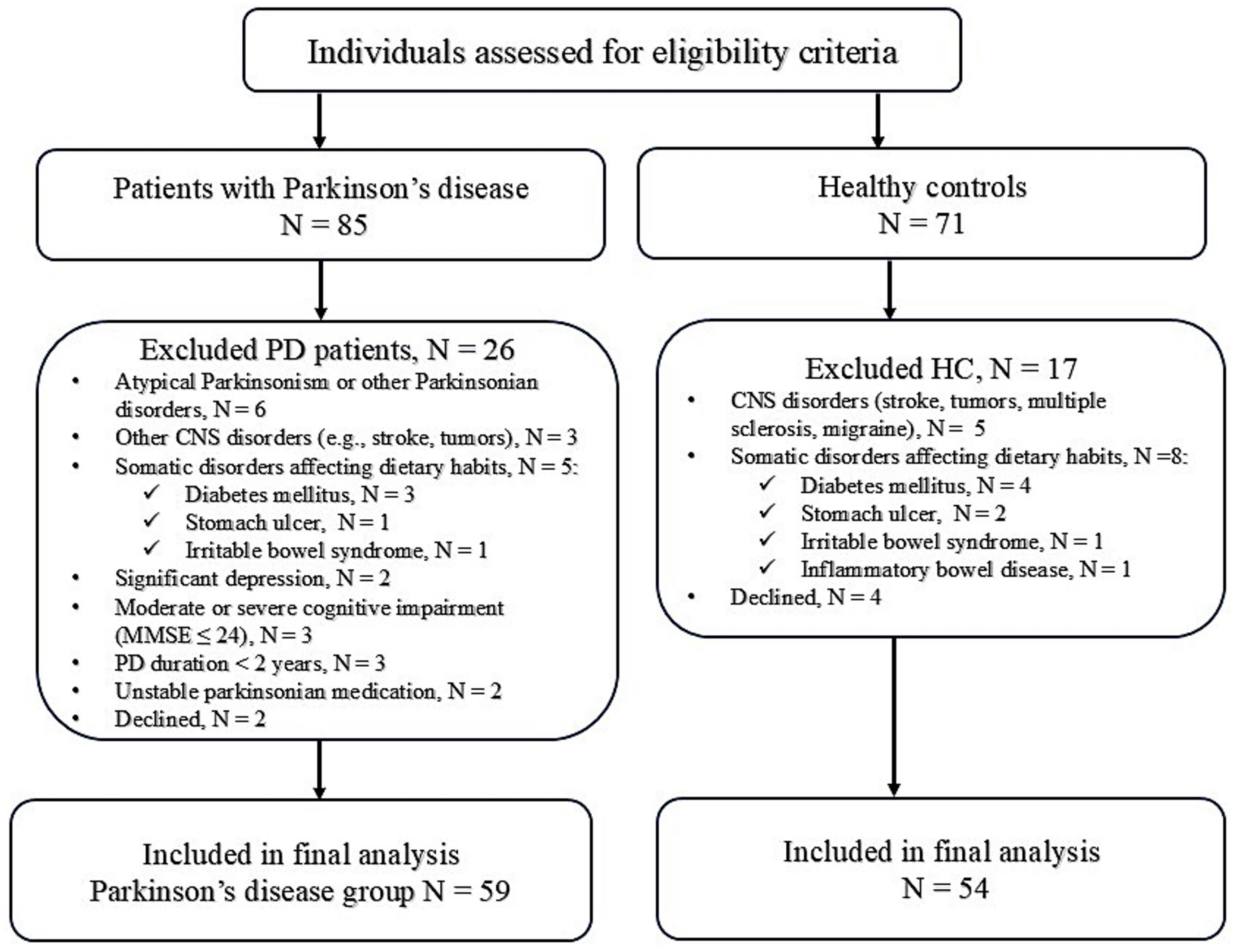
Flow diagram of participant selection.

Control participants were recruited from individuals attending the Neurology outpatient clinic for non-neurodegenerative, non-central nervous conditions and had only minor health issues, such as mechanical low back pain without neurological deficit, lumbar or cervical radiculopathy due to degenerative spine disease without paresis, or peripheral mononeuropathies, primarily ulnar or median nerve entrapment syndromes. Participants were excluded if they had any central nervous system disorders or somatic conditions that could affect their dietary habits and nutritional intake (e.g., diabetes mellitus, inflammatory bowel disease, irritable bowel syndrome, food intolerance). Household members of PD patients were excluded to avoid excessive matching on dietary and lifestyle factors. Detailed information on participant selection is provided in Figure 1.

This study was approved by the Vilnius Regional Bioethics Committee (Approval Number 2022/6-1,434-910). All participants agreed to participate in the study, were informed of the study procedures, and provided written informed consent by signing the relevant written informed consent form.

### 2.2 Data collection and tools

All participants provided information about their social and demographic characteristics (age, sex, occupation, marital status, income, smoking status, physical activity), as well as details about their health or disease status (comorbidities, PD duration, medication use). The age of PD onset was defined as the age at which the participant first began to experience motor symptoms. The daily dose of pharmacological therapy was estimated using the levodopa equivalent dose calculation (LED) (38). Physical activity (PA) was assessed using a short version of the International Physical Activity Questionnaire (IPAQ). The recorded physical activity was quantified in metabolic equivalent minutes (MET) per week for each type of PA, and the overall MET was computed by adding these values together. Subsequently, the data obtained were processed, adhering to the guidelines set by the IPAQ, and the total MET-min/week score was classified into three levels of low (< 600 MET-minutes/week), moderate (600–3,000 MET-minutes/week), and high (> 3,000 MET-minutes/week) physical activity (39). Body weight and height were measured with the individual wearing light indoor clothing and no shoes. Body mass index (BMI) was calculated by dividing weight (kg) by height (m^2^). The World Health Organization criteria for obesity were used to classify BMI levels: underweight (BMI < 18.5), healthy weight (BMI = 18.6–24.9), overweight (BMI = 25–29.9), and obesity (BMI > 30) (40).

### 2.3 Assessments of motor and non-motor symptoms

A neurologist with experience in movement disorders examined all participants. PD motor features were assessed using the Movement Disorder Society-Unified Parkinson’s Disease Rating Scale (MDS-UPDRS) from part I to part IV, and disease severity by the Hoehn-Yahr staging system (41). Participants were asked to take their usual medications and were evaluated during the best “ON” period of their medication. The Epworth Sleepiness Scale (ESS) was used to assess daytime sleepiness (EDS) (42). Consistent with the other studies, an ESS of 10 points or greater was set as a cutoff for EDS (43). The Parkinson’s Disease Sleep Scale – 2 (PDSS-2) was used to assess sleep quality, with >18 points serving as a cut-off to determine poor sleep quality (44). The Hospital Anxiety and Depression Scale (HADS) was used to assess anxiety and depression levels (45). A cut-off score ≥ 11 was used to determine clinically relevant “depressed mood,” and a cut-off score ≥ 7 was used to determine clinically relevant anxiety (46, 47). The Parkinson’s Disease Fatigue Scale (PDS-16) was used to evaluate fatigue, with a cut-off score of ≥ 3.3 indicating significant fatigue (48). Cognitive functioning was assessed using the Montreal Cognitive Assessment (MoCA), with a cut-off score of < 26 indicating cognitive impairment (49). The presence of constipation was evaluated according to the Rome IV criteria (50). The corresponding MDS-UPDRS Part I items were used to determine the presence of other non-motor symptoms, such as impulse control disorder (item 1.6), pain (item 1.9), urinary dysfunction (item 1.10), and orthostatic hypotension (item 1.12). Parkinson’s Disease Quality of Life Questionnaire – 39 (PDQ-39) was used to assess the quality of life of the participants (51).

### 2.4 Dietary habits’ assessment

All participants were asked about the key factors that determine food choices and changes in dietary habits related to PD. Since no comprehensive, validated food frequency questionnaire (FFQ) is available for the Lithuanian population, a 106-item FFQ was developed to assess participants’ usual food intake over the previous 12 months. The FFQ items were identified through a literature review of dietary habits among patients with PD. We refined the list based on team members’ insights into commonly consumed foods in Lithuania. The FFQ used in this study was designed to be pragmatic, aiming to avoid being overly long or exhausting for participants while covering the most commonly consumed foods in Lithuania and food used to derive MeDi score, such as different kinds of fruits, vegetables, legumes, fish, red meat, poultry, olive oil, whole grain bread, pasta, cereals, full-fat dairy products and alcoholic beverages. Participants were asked to estimate their average food intake over the previous 12 months, considering serving size (e.g., glass, table/tea spoon, slice, pack, or can). A researcher guided each participant through completing the questionnaire to help estimate serving size and evaluate consumption frequency. The use of dietary supplements was not assessed. This FFQ was not previously calibrated or validated in the Lithuanian population or for assessing adherence to the MeDi, nor did it allow calculation of energy and nutrient intake. Its primary purpose was only to estimate the frequency of consumption of foods, beverages, and food groups. For all dietary variables, participants were given 9 options for rating consumption frequency in defined servings, ranging from “never or less than once/month” to “more than 6 times a day.” The 106 food and beverage items from the FFQ were grouped into 33 distinct food groups. Using the serving size, the frequency of consumption was converted to grams per day or servings per month. The diet analysis software was not available for the constructed FFQ. Due to the small number of consumers in the food groups of sweetened fizzy drinks, decaffeinated coffee, non-alcoholic beer, and spirits, the statistical analysis was deemed meaningless; thus, these groups were removed from further analysis. We followed the method described by Panagiotakos et al. to construct the Mediterranean Diet (MeDi) score (52). The components of the MeDi score were derived from the FFQ, and average monthly consumption proportions were calculated. The MeDi score was calculated based on 11 components: fruits, vegetables (excluding potatoes), legumes, olive oil, fish, whole grains (cereals, bread, pasta), and potatoes. For these items with high consumption, a score of 5 was assigned; for low consumption, a score of 0 was assigned. A score of 5 was assigned for not consuming red and processed meat, poultry, and full-fat dairy products (cheese, yoghurt, milk), and a score of 0 for high consumption. Intermediate consumption was assigned scores of 1–4 accordingly. For alcohol consumption, ethanol intake (g/day) was estimated from the FFQ items. Alcohol intake scoring had an inverted U shape: 0 points were assigned to those consuming 0 or > 84 mL/day of ethanol, 5 points to those with consumption < 36 mL/day, and 1–4 points to higher consumption. The total score was determined by summing the scores of each component, ranging from 0 to 55 (52). The detailed scoring method for the MeDi score with cut-offs is provided in Supplementary Table 1. For this study, the MeDi score was used either as a continuous variable or categorized into tertiles (T1-T3) with T3 corresponding to better MeDi adherence.

A researcher guided each participant through the completion of the questionnaires.

### 2.5 Data analysis

Statistical analysis was performed using IBM SPSS Statistics version 26.0 (IBM Corp., Armonk, NY, United States) and R 4.5.2 (R Core Team, Vienna, Austria). Due to our use of forced completion across all the items, there was no missing data.

The primary outcome of this case–control study was PD status (PD vs. HC), with associations expressed as ORs per unit increase in the MeDi score. In additional analyses, MeDi adherence was categorized into tertiles. Prespecified secondary outcomes included the associations of selected non-motor symptoms (cognitive impairment, excessive daytime sleepiness, sleep problems, fatigue, anxiety, depressive mood, impulse control disorder, orthostatic hypotension, urinary dysfunction, constipation, and pain), which were analyzed in relation to MeDi adherence using logistic regression models.

The normality of data distribution was tested using the Shapiro–Wilk test. Normally distributed continuous variables are expressed as mean ± SD, and non-normally distributed continuous variables are expressed as median and interquartile range (IQR). Categorical variables are expressed as a percentage. Differences between the two groups were analyzed using a Student’s *t*-test for normally distributed variables and a Mann–Whitney U test for non-normally distributed continuous variables. The Kruskal-Wallis test was used for comparison between the three groups. Differences between groups with categorical variables were analyzed using a two-tailed chi-square test. Linear regression analysis was conducted to investigate the association between the MeDi score and its components, as well as the MeDi score and age at onset of motor symptoms. Binary logistic regression was employed to assess the OR of PD and non-motor symptoms in relation to the MeDi score. The confounding factors were chosen based on the literature. A value of *p* < 0.05 was considered statistically significant.

To assess the robustness of the findings, several sensitivity analyses were performed. First, an analysis with the exclusion of extreme alcohol consumers was performed to examine the effect of alcohol. Second, we repeated all analyses after recalculating the MeDi score without the alcohol component (mMeDi). Additionally, we further adjusted all models for coffee consumption. The same modeling strategy used in the primary analyses was applied to all sensitivity analyses.

## 3 Results

### 3.1 Demographic and disease-related characteristics

Fifty-nine PD patients and 54 HCs were recruited. The mean age of PD participants at dietary assessment was 67.03 ± 6.78 years, with 33 (55.9%) males in the PD group and 27 (50%) in the HC group. There were no differences between the PD and HC groups in terms of age, gender, or duration of education. The subjects in the control group had a higher employment rate (*p* = 0.002) and higher incomes (*p* = 0.002). However, the subjective assessment of financial status did not differ between the groups (*p* = 0.077). A detailed overview of the demographic characteristics of the study population is shown in Table 1.

**Table 1:** Demographic characteristics of the study population.

Variables	PD, *N* = 59	HC, *N* = 54	*p* value
Age, years	67.03 ± 6.78	65.72 ± 7.43	0.329
Male, *n* (%)	33 (55.9%)	27 (50%)	0.528
Marital status, *n* (%)			0.849
Married/living with a partner	45 (76.3%)	42 (77.8%)	
Single/divorced/widower	14 (23.7%)	12 (22.2%)	
Education, years	16.0 (13–17)	14.5 (13–16)	0.072
Education level, *n* (%)			**0.015**
Secondary	6 (10.2%)	4 (7.4%)	
College	14 (23.7%)	27 (50%)	
University	39 (66.1%)	23 (42.6%)	
Residency, *n* (%)			0.476
City	56 (94.9%)	49 (90.7%)	
Rural	3 (5.1%)	5 (9.3%)	
Employed, *n* (%)	21 (35.6%)	35 (64.8%)	**0.002**
Perceived financial status, *n* (%)			0.077
Good	12 (20.3%)	21 (38.9%)	
Average	44 (74.6%)	32 (59.3%)	
Difficult	3 (5.1%)	1 (1.9%)	
Income, €/month, *n* (%)			**0.002**
<1,000	35 (59.3%)	16 (29.6%)	
>1,000	24 (40.7%)	38 (70.4%)	
Smoking status, *n* (%)			**0.041**
Never smoked	39 (66.1%)	25 (46.3%)	
Prior smoker	13 (22.0%)	13 (24.1%)	
Current smoker	7 (11.9%)	16 (29.6%)	
Regular physical activity, *n* (%)	23 (39.0%)	12 (22.2%)	0.054
Frequency of physical activity, *n* (%)			**0.008**
Daily	15 (65.2%)	1 (8.3%)	
2–3 times per week	7 (30.4%)	8 (66.7%)	
1 time per week	1 (4.4%)	3 (25%)	
IPAQ levels, *n* (%)			0.285
Low level (< 600 MET-minutes/week)	5 (8.5%)	4 (7.4%)	
Moderate level (600–3,000 MET-minutes/week)	31 (52.5%)	21 (38.9%)	
High level (> 3,000 MET-minutes/week)	23 (39.0%)	29 (53.7%)	
BMI, kg/m^2^	27.47 ± 4.26	28.41 ± 3.94	0.226
BMI levels			0.537
Underweight (BMI < 18.5)	1 (1.7%)	0	
Healthy Weight (BMI = 18.6–24.9)	15 (25.4%)	11 (20.4%)	
Overweight (BMI = 25–29.9)	28 (47.5%)	24 (44.5%)	
Obesity (BMI > 30)	15 (25.4%)	19 (35.2%)	

Values are given as mean ± standard deviation, median (interquartile range), or number (percentage). BMI, body mass index; IPAQ, international physical activity questionnaire; HC, healthy controls; MET, metabolic equivalent; PD, Parkinson’s disease. The bold values indicate statistical significance.

The mean age of the PD onset was 59.58 ± 6.84 years, the median disease duration was 6 years (3–11), the median Hoehn-Yahr stage was 2 (2, 3), with the majority of respondents (64.4%) having Hoehn-Yahr stage II, and the mean MDS-UPDRS III score was 29.98 ± 12.61 points, ranging from 10 to 55 points. All participants were receiving antiparkinsonian medications: 47 (79.8%) were on levodopa, and 51 (86.4%) were on dopamine agonists. The median LED was 880 (532–1,250) mg. Based on the MDS-UPDRS Part I, 39 (66.1%) had orthostatic hypotension, 47 (79.7%) had urinary dysfunction, 12 (20.3%) had impulse control disorder, and 45 (76.3%) had pain. Hyposmia was reported by 32 (54.2%), and constipation was determined in 39 (66.1%) patients. Table 2 shows the disease-related characteristics of PD participants.

**Table 2:** Disease-related characteristics of the PD group.

Variable	Value
Age at diagnosis, years	59.58 ± 6.84
Disease duration, years	6.00 (3–11)
Hoehn-Yahr stage, *n* (%)	
I	5 (8.5%)
II	38 (64.4%)
III	14 (23.7%)
IV	2 (3.4%)
MDS – UPDRS part III, score	29.98 ± 12.61
PD medications	
Number of PD medications	3.00 (3–4)
LED, mg	880 (532–1,250)
Dopamine agonists’ LED, mg	240 (120–320)
Oral levodopa, *n* (%)	47 (79.7%)
Dopamine agonists, *n* (%)	51 (86.4%)
Amantadine, *n* (%)	30 (50.8%)
MAO-B inhibitors, *n* (%)	51 (86.4%)
COMT inhibitors, *n* (%)	17 (28.8%)
PDQ-39, summary index	20.73 (7.39–34.58)
Non-motor symptoms	
Cognitive impairment (MoCA ≤ 26), *n* (%)	30 (50.8%)
Excessive daytime sleepiness (ESS > 10)	13 (22%)
Sleep problems (PDSS-2 > 18)	23 (39%)
Fatigue (PFS ≥ 3.33)	20 (33.9%)
Anxiety (HAD-A ≥ 7)	20 (33.9%)
Depressive mood (HAD-D ≥ 11)	11 (18.6%)
Constipation (Roma IV) *n* (%)	39 (66.1%)
Orthostatic hypotension, *n* (%)	39 (66.1%)
Urinary dysfunction, *n* (%)	47 (79.7%)
Pain, *n* (%)	45 (76.3%)
Impulse control disorders, *n* (%)	12 (20.3%)
Hyposmia, *n* (%)	32 (54.2%)
Comorbidities	
Arterial hypertension, *n* (%)	30 (58.8%)
Cardiovascular disease, *n* (%)	3 (5.1%)
Osteoporosis, *n* (%)	3 (5.1%)
Hypothyroidism, *n* (%)	4 (6.8%)
Dyslipidemia, *n* (%)	17 (28.8%)
Gout, *n* (%)	4 (6.8%)

Values are given as mean ± standard deviation, median (interquartile range), or number (percentage). MDS-UPDRS, Movement Disorders Society Unified Parkinson’s Disease Rating Scale, PD; Parkinson’s disease; LED, levodopa equivalent dose; MAO-B, monoamine oxidase B; COMT, catechol-O-methyltransferase; MoCA, Montreal cognitive assessment; ESS, Epworth Sleepiness Scale; PLMS-2, Parkinson’s Disease Sleep Scale 2; PFS - Parkinson Fatigue Scale; HAD-A, Hospital Anxiety and Depression Scale Anxiety Domain; HAD-D, Hospital Anxiety and Depression Scale Depression Domain; PDQ-39, Parkinson’s disease Questionnaire-39.

### 3.2 Dietary habits’ characteristics

The primary factor influencing food choice for both the PD and HC groups was “taste” (respectively 54.2 and 53.7%), while “health benefits” were in third place after the “influence of family members” among PD patients and in second place among HC respondents (respectively 16.9 and 25.9%). There were no statistically significant differences in selection criteria between the two groups (*p* = 0.494). However, only half of the respondents in both groups believed their diet was healthy (32 (54.2%) in the PD and 31 (57.1%) in the HC group).

Thirty-two (54.2%) patients with PD reported that their dietary habits had changed due to the disease: 10 (31.3%) had to adjust their meal times, 11 (34.4%) changed the composition, and 4 (12.5%) changed the structure of their meals. The primary reason for dietary changes included the need to avoid food proteins and medication interactions [10 (31.3%)], and the decreased ability to prepare meals (5 (15.6%)). Twenty-one (35.6%) PD patients reported experiencing problems during cooking. Symptoms that particularly impacted meal preparation were rigidity (8 (38.1%)), followed by tremor (6 (28.6%)) and slowness (6 (28.6%)). However, most PD patients (56 (94.9%)) prepared their own meals, and only a minority bought already prepared or semi-finished meals. Additionally, more PD patients than controls usually purchased organically grown foods or foods from local farmers (respectively 19 (32.2%) vs. 8 (11.1%), *p* = 0.039).

PD patients compared to the HC, consumed statistically significantly more fruits (*p* = 0.013), vegetables (*p* = 0.045), poultry (*p* = 0.004), cereals (*p* = 0.017), black tea (*p* = 0.008), jam (*p* = 0.000), honey (*p* = 0.016) and highly processed foods (*p* = 0.024). In contrast, PD patients consumed statistically significantly fewer potatoes (*p* = 0.009) and caffeinated coffee (*p* = 0.001). The distribution of consumption of the main food groups between the PD and HC groups is presented in Supplementary Table 2.

### 3.3 The adherence to the Mediterranean diet

The MeDi score in the HC group was slightly higher than in the PD group (31.70 vs. 29.62, *p* = 0.058), but this difference did not reach statistical significance. No sociodemographic factors were related to MeDi scores in the PD group. Still, those with higher educational levels and higher incomes had higher MeDi scores in the control group, respectively, 32.32 ± 3.83 points vs. 34.30 ± 4.96, *p* = 0.005 for secondary vs. university education, and 29.38 ± 5.76 and 32.76 ± 4.78, *p* = 0.030 for lower vs. higher income groups. Regarding MeDi components, respondents in the PD group consumed less potatoes [12.00 (4.00–14.00) vs. 13.00 (11.00–24.00), *p* = 0.009) and alcohol (7.00 (0.00–13.00) vs. 13 (0.00–40.00), *p* = 0.016] and more fruits [70.00 (44.00–102.00) vs. 54.00 (35.00–88.00), *p* = 0.012), poultry (12 (12.00–16.00) vs. 8.00 (4.00–14.00), *p* = 0.004], and olive oil (5.5 (3.00–75) vs. 3.00 (0.5–6.00), *p* = 0.024). Detailed information on MeDi components in the PD and HC groups is provided in Table 3. The following food groups were associated with a higher MeDi score in the PD group (Adjusted R^2^ = 0.539, ANOVA *p* = 0.000): whole grain products (*β* = 0.234, *p* = 0.033), potatoes (*β* = 0.231, *p* = 0.024), legumes (*β* = 0.265, 0.008), fish (*β* = 0.383, *p* = 0.001), olive oil (*β* = 0.260, *p* = 0.015). In the HC group (Adjusted R^2^ = 0.632, *p* = 0.000): whole grain products (*β* = 0.231, *p* = 0.026), legumes (*β* = 0.302, *p* = 0.014), fish (*β* = 0.248, *p* = 0.014), olive oil (*β* = 0.348, *p* = 0.002). Conversely, red meat and its products (*β* = −0.250, *p* = 0.017) were associated with a lower MeDi score in the HC group (Supplementary Table 3).

**Table 3:** Frequency of consumption of MeDi score components in PD and HC groups.

MeDi score components, servings/month	PD, *N* = 59	HC, *N* = 54	*p* value
MeDi score, points	29.62 ± 5.45	31.70 ± 5.24	0.058
Non-refined cereals, bread, pasta	22.00 (10.00–34.00)	18 (7.50–34.50)	0.559
Potatoes	12.00 (4.00–14.00)	13.00 (11.00–24.00)	**0.009**
Fruits	70 (44.00–102.00)	54 (35.00–88.00)	**0.012**
Vegetables	76 (56.00–108.00)	68 (48.00–92.25)	0.138
Legumes	6.00 (4.00–8.00)	4.00 (2.00–6.00)	0.062
Fish	6.00 (6.00–12.00)	8.00 (4.00–14.00)	0.128
Red meat and products	23.5 (13.00–36.00)	19.25 (10.36–31.00)	0.243
Poultry	12.00 (12.00–16.00)	8.00 (4.00–14.00)	**0.004**
Full fat dairy products (cheese, yoghurt, milk)	35.5 (19.25–54.00)	31.5 (11.81–45.75)	0.12
Use of olive oil in cooking (times/week)	5.5 (3.00–7.5)	3.00 (0.5–6.00)	**0.024**
Alcohol (g/d of ethanol)	7.00 (0–13.00)	13.00 (0–40.00)	**0.016**

Values are given as mean ± standard deviation, median (interquartile range). HC, healthy controls; MeDi, Mediterranean diet; PD, Parkinson’s disease. The bold values indicate statistical significance.

#### 3.3.1 The association between adherence to the Mediterranean diet and Parkinson’s disease status

The association between MeDi adherence and PD status was not significant in the crude model (OR per 1-point increase = 0.93, 95% CI 0.87–1.00, *p* = 0.063). After adjustment for age and gender, a borderline inverse association with lower PD odds was observed (OR per 1-point increase = 0.93, 95% CI 0.86–0.99, *p* = 0.050). A statistically significant association with lower PD odds emerged in the models further adjusted for smoking status, BMI, and physical activity (OR per 1-point increase = 0.92, 95% CI 0.85–0.99, *p* = 0.042), as well as in the fully adjusted model that also included education and income (OR per 1-point increase = 0.90, 95% CI 0.81–0.98, *p* = 0.025). When comparing PD odds across MeDi tertiles, a statistically significant reduction in PD odds was consistently observed for T3 across all models (Table 4).

**Table 4:** The association between PD status and Mediterranean diet adherence.

Model	MeDi score continuous			MeDi score tertiles				
	OR	95% CI	*p* value	Tertile (ref: T1)	OR	95% CI	*p* value	*p* for trend
Univariate model	0.93	0.87; 1.00	0.063	T2 vs. T1	0.39	0.15; 0.94	**0.038**	**0.048**
				T3 vs. T1	0.40	0.15; 0.99	0.050	
Model 1	0.93	0.86; 0.99	0.050	T2 vs. T1	0.39	0.15; 0.97	**0.046**	**0.037**
				T3 versus T1	0.37	0.14; 0.95	**0.040**	
Model 2	0.92	0.85; 0.99	**0.042**	T2 vs. T1	0.45	0.16; 1.21	0.117	**0.027**
				T3 versus T1	0.31	0.10; 0.87	**0.029**	
Model 3	0.90	0.81; 0.98	**0.025**	T2 vs. T1	0.37	0.11; 1.14	0.087	**0.009**
				T3 versus T1	0.19	0.05; 0.65	**0.010**	

Model 1: MeDi score, age, gender; Model 2: MeDi score, age, gender, smoking status, body mass index, physical activity; Model 3: MeDi score, age, gender, smoking status, body mass index, physical activity, education, income. The bold values indicate statistical significance.

When we repeated all analyses after recalculating the MeDi score without the alcohol component (mMeDi), the results were comparable to the primary analysis, with similar effect estimates observed (Supplementary Table 4). After excluding participants with extreme alcohol consumption, the direction and magnitude of the association between MeDi adherence and PD status were similar to the primary analysis; however, most estimates did not reach statistical significance. A statistically significant inverse association was observed only in the fully adjusted model when MeDi was analyzed in tertiles (*p* for trend = 0.033). The detailed results are presented in Supplementary Table 5. Further adjustment for coffee consumption yielded similar effect estimates, although most associations were not statistically significant, except for the fully adjusted tertile model (*p* for trend = 0.023) (Supplementary Table 6).

#### 3.3.2 The association between adherence to the Mediterranean diet and PD motor and non-motor symptoms

There was no statistically significant association between MeDi score and age at motor symptom onset in either the crude or adjusted models for continuous MeDi score. When MeDi adherence was analyzed by tertiles, participants in the highest tertile (T3) had a later age at motor symptom onset compared to those in the lowest tertile (B = 4.06, 95% CI 0.17–7.94, *p* = 0.041). However, this association attenuated and was no longer statistically significant after adjustment for additional confounding factors (age, gender, smoking status, body mass index, physical activity, education, income).

There was no statistically significant association between the continuous MeDi score and LED in either the crude or adjusted models (adjusted for gender, disease duration, and MDS-UPDRS part III score). In tertile-based analyses, participants in T3 had a lower LED than those in T1 (B = −317.0, 95% CI -599.0, −34.7, *p* = 0.029). This association, however, was no longer statistically significant after adjustment for additional confounding factors (gender, disease duration, MDS-UPDRS part III score).

We did not find any statistically significant association between MeDi score (as a continuous variable and by tertiles) and motor symptoms assessed by the MDS-UPDRS part III, either in crude models or after adjustment for potential confounders. A higher MeDi score was associated with lower odds of anxiety (OR per 1-point increase = 0.88, CI 95% 0.78–0.98, *p* = 0.028), urinary dysfunction (OR per 1-point increase = 0.86, CI 95% 0.74–0.98, *p* = 0.029), and constipation (OR per 1-point increase = 0.88, CI 95% 0.78–0.98, *p* = 0.027) in univariate analysis. This association remained statistically significant after adjustment for age, gender, smoking status, BMI, physical activity level, education, and income. Additionally, a higher MeDi score was associated with lower odds of pain (OR per 1-point increase = 0.86, 95% CI 0.74–0.97, *p* = 0.022); however, this association was only observed in the unadjusted model (Supplementary Table 7). According to Parkinson’s Disease Questionnaire – 39 (PDQ-39), patients in the T3 had lower scores for emotional well-being, stigma, and body discomfort compared to those in the T1 tertile, respectively 16.22 ± 12.94 vs. 26.93 ± 19.49 (*p* = 0.035), 11.84 ± 10.12 vs. 29.46 ± 26.51 (*p* = 0.013), 27.16 ± 26.20 vs. 39.88 ± 23.28 (*p* = 0.029).

## 4 Discussion

### 4.1 Dietary habits

In the present study, we observed that patients with PD had different dietary habits and food preferences compared to controls. In our study, 32.9% PD patients reported changing their dietary habits due to the disease. This is lower than the 55.4% reported by Baert et al. Such a discrepancy may be attributed to more severe disease symptoms in their study. Although Baert and co-authors did not provide information on Hoehn-Yahr staging or the severity of motor symptoms, even 21.1% of individuals in their cohort reported reduced ability to cook, 60.7% experienced problems during cooking, and only 39.7% of respondents were responsible for meal preparation (53). In contrast, our study showed that only 35.6% of PD patients reported problems with cooking, while 94.9% prepared meals themselves.

In the present study, we found that PD patients consumed more fruits, vegetables, poultry, cereals, black tea, honey, jam, and highly processed foods, and less potatoes and caffeinated coffee, compared to HCs. Different dietary habits among patients with PD compared to controls were found by Cassani et al. In Cassani et al.’s study, patients consumed less alcohol, fish, water, coffee, and milk, and had lower total food intake. However, they consumed more fruit, cooked vegetables, cereals, baked items, dressings, and sweets (54). Kwon et al. also found lower caffeine and coffee consumption in PD patients than in controls (31). The reduced consumption of coffee and alcohol is consistent with the well-known behavioral traits of PD patients (55). Higher fruit and vegetable consumption in PD patients can be associated with the high prevalence of constipation in this population (56). The proportion of patients with constipation in our study was quite high (66.1%). Patients with constipation are usually recommended to increase their intake of fiber-rich foods such as fruits, vegetables, and grains (53). On the other hand, increased intake of sweet fruits, as well as jams and honey, may be related to patients’ tendency to consume more sugar. Several studies have reported an increased consumption of total and added sugars in PD patients (31, 57). This may serve as a compensatory mechanism for disease-related dopamine loss, as sugar increases brain dopamine through insulin and alters brain activity in reward-processing regions (58). Higher sugar consumption can also be associated with a higher dopamine agonist dosage, linked to impulse control disorders and binge eating (31, 59). It is crucial to note that dietary patterns with a high intake of fruits, vegetables, legumes, fish, nuts, and poultry can help protect against PD and slow disease progression (15, 20, 25). According to Mischeley et al. study, fresh vegetables and fruits, nuts and seeds, non-fried fish, olive oil, wine, coconut oil, and fresh herb species were associated with the reduced rate of PD progression and consumption of canned fruits and vegetables, diet and non-diet soda, fried foods, beef, ice cream, yogurt, and cheese were associated with increased disease progression (60). Binge eating and changes in personality traits may also contribute to the higher intake of ultra-processed foods among PD patients. On the other hand, high consumption of ultra-processed foods may increase the risk of prodromal non-motor symptoms of PD; however, further studies are warranted to confirm the association between such consumption and the risk of PD (61). In our study, 32.2% of patients with PD purchased foods from local farmers. This type of nutritional behavior could be associated with a lower burden of PD symptoms (60).

### 4.2 Adherence to the Mediterranean diet

In the present study, we found a trend toward greater adherence to the MeDi in the HC group than in patients with PD. Several other studies have reported no significant difference in adherence to the MeDi between patients and controls (16, 54). Conversely, Alcalay et al. found that the MeDi score of PD patients was lower than that of controls (13). This result could be attributed to the larger number of participants in their study, as well as to patients’ lifestyle, genetic factors, and environmental conditions.

To our knowledge, no studies have evaluated MeDi adherence in the general Lithuanian population or among older Lithuanians. However, a study assessing MeDi compliance in young Lithuanians (16–18 years old) showed that only 7% fully complied with the MeDi, and 1/3 had poor compliance. Female gender, higher education, and a sufficient level of physical activity predicted a healthier diet (34). According to the systematic review evaluating the adherence to the MeDi among adults in Mediterranean countries, adherence to this diet is generally low to moderate (62). The mean MeDi score in our study was 29.62 in PD patients and 31.70 in controls, corresponding to a low adherence level according to the cut-offs used in the above-mentioned review. We found no association between adherence to the MeDi and demographic variables in the PD group. However, Metcalfe-Roach et al. found that the older PD patients and those with a later age of disease onset had higher adherence to MeDi, but this pattern was observed only in men (18). According to Biggi et al., predictors of adherence to the MeDi can vary by country. However, a common predictor across almost all studied countries is a positive attitude toward the healthiness of food. Other predictors found in different countries were health motivations, weight control, and preference for local and seasonal foods (63). The lack of association between the MeDi score and demographic factors among PD patients may be due to the disease influencing dietary choices regardless of demographic indicators. According to a study conducted in young Lithuanian adults, non-compliance with MeDi was mostly related to the under-consumption of olive oil, nuts, fish, seafood, legumes, and wine, as well as over-consumption of red meat (34). In our study, a higher MeDi score was linked to increased consumption of whole grain products, potatoes, legumes, fish, and olive oil in the PD group. Conversely, a lower score was associated with higher red meat consumption, but only in the HC cohort.

#### 4.2.1 Adherence to the Mediterranean diet and odds of Parkinson’s disease

We did not observe an association between adherence to the MeDi and the PD status in a crude model. However, after adjustment for age and gender, there borderline inverse association emerged, which became statistically significant after further adjustment for smoking status, BMI, and physical activity, as well as in the fully adjusted model that additionally included education and income. When MeDi adherence was analyzed by tertiles, individuals in the highest tertile consistently demonstrated lower odds of PD across all models. These findings suggest that the relationship between MeDi adherence and PD status becomes more apparent only after accounting for key demographic and lifestyle factors. This pattern is consistent with previous studies reporting an inverse association between MeDi adherence and PD status after adjustment for potential confounders (13, 15, 27). Xu et al. observed a lower odds of PD in both crude and adjusted models (32). Other authors have noted that MeDi shows only marginal association with PD odds and disease progression (25, 33). Keramati et al. found no association between MeDi adherence and odds of PD (16).

In sensitivity analyses examining the potential influence of alcohol consumption, slightly different patterns were observed. When the MeDi score was recalculated without the alcohol component (mMeDi), the inverse association with PD status remained largely unchanged, suggesting that the observed association was not driven solely by alcohol intake. In contrast, exclusion of participants with extreme alcohol consumption led to attenuation of statistical significance in most models, although the direction and magnitude of the associations were similar to the primary analysis. This likely reflects reduced statistical power following sample size reduction rather than a meaningful change in effect. The role of alcohol consumption in PD remains considerably uncertain. A systematic review and meta-analysis by Jiménez-Jiménez et al. suggested an inverse association between alcohol consumption and PD, supported by case–control studies but not clearly by prospective ones (64). Some studies suggest potential nonlinear associations, in which moderate alcohol intake may be associated with lower PD risk, whereas heavy consumption does not confer benefit (65). Additionally, associations may differ by beverage type and geographic area (65, 66). Interpretation is further complicated by potential confounding by smoking, which is strongly inversely associated with PD risk and positively associated with alcohol consumption, as well as reverse causation (67).

In our study, additional adjustment for coffee consumption attenuated the association between MeDi adherence and PD, with most estimates no longer reaching statistical significance, although the direction and magnitude of the associations remained similar. This attenuation may reflect, at least in part, the independent contribution of caffeine to neuroprotection. A recent meta-analysis by Hong et al. reported that higher caffeine intake was associated with a reduced risk of PD and may also influence disease progression. Proposed biological mechanisms include antagonism of adenosine A2A receptors, which are highly expressed in the basal ganglia and interact with dopaminergic signaling pathways. A2A receptor blockade has been shown to modulate neuroinflammation-mediated neuronal dysfunction and degeneration (68).

#### 4.2.2 Association between adherence to the Mediterranean diet and PD motor and non-motor symptoms

We did not observe the association between adherence to MeDi and PD severity, measured by the MDS-UPDRS scale. Evidence for an association between dietary patterns and PD severity is limited. Similar results were found in a study conducted by Keramati et al. (16). On the contrary, Paknahad et al. reported a beneficial association between higher MeDi adherence and lower PD severity, as measured by the UPDRS (22). In the study conducted by Fox et al., after adjustment for age, gender, income, and years since diagnosis, for each 1-point increase in the MeDi scores, PRO-PD (Patient-Reported Outcome scale used to measure PD symptom severity) scores were found to be 25.6 points lower (14).

We found that PD patients in the highest MeDi adherence tertile had a later onset of motor symptoms; however, this association was attenuated and no longer statistically significant after adjusting for other confounders. Nevertheless, several studies have reported that higher MeDi scores were associated with a later onset of motor symptoms after adjustment for confounders (13, 18).

We found that higher adherence to the MeDi was associated with lower odds of anxiety, pain, constipation, and urinary dysfunction. Previous studies have reported that higher MeDi adherence was associated with fewer or less severe non-motor symptoms of PD, including cognitive impairment, depression, and sleep disturbances in patients with PD (14, 21, 69). The 5-week single-arm MeDi intervention study demonstrated a decrease in constipation symptoms among patients with PD, associated with changes in gut microbiota (23). There is a limited amount of research on the MeDi association with pain in PD patients. Still, several studies have found that MeDi can improve pain in patients with fibromyalgia, osteoarthritis, and rheumatoid arthritis due to its anti-inflammatory and antioxidative properties (70–72). Additionally, we identified an association between MeDi adherence and urinary dysfunction, which has not been previously reported in patients with PD and requires further investigation. However, a study investigating the relationship between MeDi and prodromal features of PD in elderly (>65 years) PD-free individuals found that higher adherence was associated with a lower prevalence of urinary dysfunction (17). The beneficial effect of MeDi on urinary symptoms may be associated with lower systemic and local inflammation, reduced oxidative stress, and lower BMI (73–75).

We have not found an association between MeDi and quality of life, as measured by the total PDQ-39 score. However, participants with the highest MeDi adherence (T3) showed better scores for emotional well-being and stigma on the PDQ-39 compared to those with lower adherence (T1). This finding may reflect several mechanisms. MeDi adherence is often associated with healthier lifestyle behaviors, such as regular physical activity and non-smoking, as well as higher socioeconomic status and stronger social networks, all of which can support emotional well-being and reduce perceived stigma (76, 77). While similar relationships between overall diet quality and quality of life have been reported in PD, to our knowledge, no studies have directly examined MeDi adherence in relation to PDQ-39 emotional well-being or stigma subdomains (78). Therefore, our findings suggest a potential link between MeDi adherence and psychosocial aspects of PD, but further research is needed to confirm these associations and clarify underlying mechanisms.

Due to the complexity of the diets, the key elements that contribute to their beneficial effects are not well understood. MeDi-like diets may protect neurons and slow neurodegeneration through several mechanisms of action: the increased intake of polyphenols, resveratrol, omega-3 fatty acids, vitamins C and E, carotenoids from fruits and vegetables, olive oil, and red wine. These components exert anti-oxidative and anti-inflammatory effects that are important contributors to PD (7). Previous studies have shown that participants with higher adherence to MeDi have better oxidative status and lower inflammation (22, 79). Another potential mechanism is the modulation of gut microbiota by a diet high in fiber and polyphenols, which both increase the amount of beneficial bacteria (23). Several studies reported that the gut microbiome is different in PD patients compared to healthy controls, with increased abundance of pro-inflammatory microorganisms that promote neuro-inflammation and can contribute to PD pathogenesis (8, 80). The Mediterranean diet is associated with a higher intake of dietary fiber, which can relieve constipation and improve indigestion (23).

### 4.3 Limitations

Several limitations need to be considered when interpreting the results of our study. The main limitation of this study and a potential source of bias is the FFQ developed by the study team based on their experience and review of the literature. The FFQ was neither calibrated nor validated in the Lithuanian population, and for deriving the MeDi score, potentially affecting the scale’s accuracy. Furthermore, we could only gather information on the frequency of different food and beverage consumption, measured in portions per month or grams per day, without the possibility of further calculation of daily caloric, macro, and micronutrient intake. Additionally, self-reported dietary habits are notoriously difficult to assess, and it is possible that study participants were underestimating/overestimating their consumption. Moreover, PD patients may have cognitive impairment, which can affect their ability to accurately recall their dietary intake. To mitigate this limitation, patients with an MMSE score ≤ 24 were excluded from the study to mitigate this potential bias. However, using the MOCA, we found that despite the MMSE results, about 50% of patients had some cognitive dysfunction. Furthermore, FFQ was designed to assess recent rather than past dietary habits. Therefore, the relationship between the odds of developing PD and the adherence to the MeDi should be assessed with caution, as the respondents’ dietary habits may have differed decades ago, when the pathological mechanisms of the disease began, from the ones they declare now. On the other hand, non-motor symptoms such as constipation, hyposmia, anxiety, and depression may have influenced patients’ food choices and dietary habits over time. Thus, the reverse causality could be another explanation for the association between the MeDi score and non-motor symptoms. In addition, a relatively small sample size may have insufficient power to detect small effects of the MeDi on the odds of PD and disease symptoms. Another potential bias is recruiting controls with pain-related conditions as they can induce sleep disturbances and potentially influence lifestyle factors such as coffee and alcohol consumption to counteract fatigue or daytime sleepiness or to reduce pain (81–84). On the other hand, persons with pain-related conditions can diminish alcohol consumption to avoid alcohol interaction with medications. To eliminate selection bias, people were randomly selected after visiting our clinic. However, there is still a possibility that those who consent to participate in this survey may be more health-conscious and potentially have a greater adherence to a healthy diet. Additionally, the observational design of the study limits the interpretation of causality.

Our study also has several strengths. We used *a priori* MeDi score, rather than a population-specific one. Multiple MeDi adherence scores have been proposed, differing in structure, scoring ranges, and assumptions about dietary distributions, which may limit direct comparability across studies, particularly in non-Mediterranean populations. The early MeDi scoring systems (i.e., Trichopoulou’s MeDi Scale and the alternative MeDi scale) are widely used but employ population-specific median cut-offs, which limit interpretation and cross-study comparisons (85). *A priori* dietary scores are based on predefined intake frequencies and fixed cut-offs, reducing reliance on local distributions of dietary intakes and enabling comparison with other populations in similar studies. Also, the scale proposed by Panagiotakos et al. was used in various dietary studies involving patients with PD in non-Mediterranean countries (18, 33). A movement disorder specialist performed the diagnosis of PD and evaluation of motor and non-motor symptoms, which helped to exclude other etiologies of Parkinsonism and made the evaluation of symptoms more reliable. Moreover, we have used validated scales to assess motor and non-motor symptoms in PD patients. Additionally, we have adjusted our analysis to account for multiple potential confounders, including demographic and clinical characteristics.

## 5 Conclusion

Our results suggest that patients with PD have different dietary habits and food preferences compared to controls, and the disease has an influence on the dietary habits of PD patients. However, adherence to the MeDi diet has been found to be low in the elderly Lithuanian population (> 65 years), including patients with PD. Higher adherence to the MeDi was associated with lower odds of PD after adjustment for potential confounders, as well as lower odds of selected non-motor symptoms. The MeDi may be a potential modifiable lifestyle factor to improve PD symptoms. Therefore, it is crucial to enhance patients’ understanding of the significance of a Mediterranean diet (MeDi) and other healthy diets, the main components that contribute to them, their potential mechanisms of action, and the positive benefits they may offer for the course of PD and the reduction of its symptoms.

## Data Availability

The raw data supporting the conclusions of this article will be made available by the authors, without undue reservation.
