# Supplementary material for: Dietary habits and adherence to the Mediterranean diet in a cohort of Parkinson’s disease patients in Lithuania
Source: Front Nutr. 2026 Mar 9;13:1773331. doi: 10.3389/fnut.2026.1773331 (PMC13006276; doi:10.3389/fnut.2026.1773331)
Supplement: Supplementary file 1 [file Table_1.docx]

Supplementary Table 1. Scoring method for the MeDi score.

|  | Frequency of consumption (servings/month) | | | | | |
| --- | --- | --- | --- | --- | --- | --- |
| **Points** | **0** | **1** | **2** | **3** | **4** | **5** |
| Non-refined cereals, bread, pasta | Never | 1-4 | 5-8 | 9-12 | 13-18 | >18 |
| Potatoes | Never | 1-4 | 5-8 | 9-12 | 13-18 | >18 |
| Fruits | Never | 1-4 | 5-8 | 9-12 | 13-18 | >18 |
| Vegetables (not potatoes) | Never | 1-4 | 5-8 | 9-12 | 13-18 | >18 |
| Legumes | Never | 1-4 | 5-8 | 9-12 | 13-18 | >18 |
| Fish | Never | 1-4 | 5-8 | 9-12 | 13-18 | >18 |
| Olive oil | Never | Rarely | 1 | 2-3 | 4-5 | Daily |
| **Points** | **5** | **4** | **3** | **2** | **1** | **0** |
| Red meat and products | Never | 1-4 | 5-8 | 9-12 | 13-18 | >18 |
| Poultry | Never | 1-4 | 5-8 | 9-12 | 13-18 | >18 |
| Full-fat dairy products (cheese, yoghurt, milk) | Never | 1-4 | 5-8 | 9-12 | 13-18 | >18 |
| Alcohol (g/d of ethanol) | <36 | 36-48 | 48-60 | 60-72 | 72-84 | >84 or 0 |

Supplementary Table 2. Frequency of consumption for different food groups in the PD and HC groups

| **Food group** | **PD, N=59** | **HC, N= 54** | **p-value** |
| --- | --- | --- | --- |
| Dairy products, g/day | 165.33 (114.00-266.67) | 152.67 (71.25-225.50) | 0.132 |
| Fermented dairy products, g/day | 80.00 (26.67-146.67) | 80.00 (24.17-130.00) | 0.599 |
| Fruits, g/day | 329.00 (191.5-462.23) | 223.00 (151.33-397.33) | **0.013** |
| Berries, g/day | 13.33 (11.67-40.00) | 26.67 (13.33-40.00) | 0.959 |
| Vegetables, g/day | 269.33 (203.67-347.00) | 240.00 (179.00-307.5) | **0.045** |
| Potato, g/day | 40.00 (13.33-46.67) | 43,33 (36.67-80.00) | **0.009** |
| Legumes, g/day | 20.00 (13.33-26.67) | 13.33 (6.67-20.00) | 0.090 |
| Fermented vegetables, g/day | 8.27 (4.93-21.60) | 8.27 (4.90-27.80) | 0.635 |
| Read meat, g/day | 46.67 (20.00-53.33) | 33.33 (13.33-53.33) | 0.138 |
| Poultry, g/day | 40.00 (40.00-53.33) | 26.6 (13.33-46.67) | **0.004** |
| Processed meat, g/day | 8.33 (1.33-25.83) | 11.33 (4.33-29.33) | 0.261 |
| Fish, g/day | 26.67 (13.33-41.67) | 30.00 (20.00-46.67) | 0.153 |
| Grains, g/day | 60.00 (40.00-93.33) | 40.00 (20.00-73.33) | **0.017** |
| Bread, pasta, g/day | 108.67 (62.00-136.67) | 60.67 (45.00-119.33) | 0.061 |
| Eggs, g/day | 20.00 (20.00-36.67) | 20.00 (20.00-36.67) | 0.712 |
| Sweetened drinks, ml/day | 13.33 (0.00- 80.00) | 13.33 (00.0-40.00) | 0.059 |
| Sweets, ml/day | 8.67 (0.5-28.33) | 7.67 (1.00-12.67) | 0.258 |
| Pastry, g/day | 20.00 (9.17-52.00) | 12.00 (8.00-48.33) | 0.092 |
| Black tea, ml/day | 26.67 (0.00-200.00) | 13.33 (0.00-80.00) | **0.008** |
| Green tea, ml/day | 13.33 (0.00-80.00) | 13.33 (0.00-26.67) | 0.602 |
| Herbal tea, ml/day | 26.67 (0.00-80.00) | 13.33 (0.00- 96.67) | 0.608 |
| Caffeinated coffee, ml/day | 200.00 (146.67-200.00) | 400.00 (200.00-400.00) | **0.001** |
| Alcoholic beer, ml/day | 0 (0.00- 33.33) | 0 (0.00-33.33) | 0.207 |
| Wine, ml/day | 0 (0.00-6.67) | 6.67 (0.00-8.33) | 0.302 |
| Jam, g/day | 4.00 (3.33-20.00) | 0.67 (0.00-4.00) | **<0.001** |
| Honney, g/day | 4.00 (0.67-10.00) | 0.67 (0.00-4.00) | **0.016** |
| Added sugar, g/day | 2.00 (0.00-5.00) | 0.00 (0.00-5.00) | 0.150 |
| Nuts, g/day | 6.67 (2.5-20.00) | 6.67 (0.00-20.00) | 0.628 |
| Ultra-processed food, g/day | 172.5 (113.00-229.00) | 121.25 (78.87-181.75) | **0.024** |
| Water, glasses/day | 3.00 (2.00-5.00) | 4.00 (3.00-5.00) | 0.904 |

Values are given as median (interquartile range). HC, healthy controls; PD, Parkinson’s disease. The bold values indicate statistical significance.

Supplementary Table 3. Association of the Mediterranean diet score and its components in the PD and HC groups.

| **MeDi index components, servings/month** | **PD**  p^1^ = 0.000  R^2^ = 0.539 | | **HC**  p^1^ = 0.000  R^2^ = 0.632 | |
| --- | --- | --- | --- | --- |
|  | **β** | p-value | **β** | p-value |
| Non-refined cereals, bread, pasta | 0.234 | **0.033** | 0.231 | **0.026** |
| Potatoes | 0.231 | **0.024** | 0.140 | 0.186 |
| Fruits | -0.054 | 0.591 | -0.018 | 0.853 |
| Vegetables | 0.125 | 0.290 | -0.015 | 0.876 |
| Legumes | 0.265 | **0.008** | 0.302 | **0.002** |
| Fish | 0.383 | **0.001** | 0.248 | **0.014** |
| Red meat and products | -0.126 | 0.248 | -0.250 | **0.017** |
| Poultry | -0.149 | 0.167 | -0.167 | 0.073 |
| Full fat dairy products (cheese, yoghurt, milk) | -0.022 | 0.843 | -0.092 | 0.325 |
| Use of olive oil in cooking (times/week) | 0.260 | **0.015** | 0.348 | **0.002** |
| Alcohol (ml/d of ethanol) | 0.193 | 0.094 | 0.161 | 0.116 |

The bold values indicate statistical significance.

Supplementary Table 4. Asociation between PD status and Mediterranean diet adherence using a modified MeDi (mMeDi) score (excluding the alcohol component).

|  | mMeDi score continuous | | | mMeDi score tertiles | | | | |
| --- | --- | --- | --- | --- | --- | --- | --- | --- |
|  | OR | 95% CI | p value |  | OR | 95% CI | p value | p for trend |
| Univariate model | 0.95 | (0.87; 1.03) | 0.230 | T2 vs. T1 | 0.38 | (0.15; 0.94) | 0.038 | **0.040** |
|  |  |  |  | T3 vs. T1 | 0.39 | (0.15; 0.99) | 0.051 |  |
| Model 1 | 0.95 | (0.81;1.03) | 0.227 | T2 vs. T1 | 0.39 | (0.15; 0.97) | **0.046** | **0.031** |
|  |  |  |  | T3 vs. T1 | 0.37 | (0.14; 0.95) | **0.041** |  |
| Model 2 | 0.92 | (0.84; 1.02) | 0.100 | T2 vs. T1 | 0.45 | (0.16; 1.21) | 0.117 | **0.025** |
|  |  |  |  | T3 vs. T1 | 0.31 | (0.10; 0.87) | **0.029** |  |
| Model 3 | 0.91 | (0.81; 1.01) | 0.071 | T2 vs. T1 | 0.42 | (0.13; 1.30) | 0.136 | **0.009** |
|  |  |  |  | T3 vs. T1 | 0.18 | (0.05; 0.62) | **0.009** |  |

Model 1: mMeDi score, disease duration, age, gender; Model 2: mMeDi score, disease duration, age, gender, physical activity; Model 3: mMeDi score, disease duration, age, gender, physical activity, income. The bold values indicate statistical significance.

Supplementary Table 5. Association between PD status and Mediterranean diet adherence after exclusion of extreme alcohol consumers (PD, n = 57; HC, n = 48).

|  | MeDi score continuous | | | MeDi score tertiles | | | | |
| --- | --- | --- | --- | --- | --- | --- | --- | --- |
|  | OR | 95% CI | p value |  | OR | 95% CI | p value | p for trend |
| Univariate model | 0.95 | (0.88; 1.03) | 0.194 | T2 vs. T1 | 0.41 | (0.16; 1.03) | 0.061 | 0.159 |
|  |  |  |  | T3 vs. T1 | 0.52 | (0.19; 1.38) | 0.190 |  |
| Model 1 | 0.95 | (0.88; 1.02) | 0.164 | T2 vs. T1 | 0.43 | (0.17; 1.07) | 0.072 | 0.137 |
|  |  |  |  | T3 vs. T1 | 0.49 | (0.18; 1.33) |  |  |
| Model 2 | 0.92 | (0.85; 1.01) | 0.073 | T2 vs. T1 | 0.48 | (0.17; 1.31) | 0.155 | 0.104 |
|  |  |  |  | T3 vs. T1 | 0.42 | (0.14; 1.23) | 0.117 |  |
| Model 3 | 0.91 | (0.83; 1.01) | 0.063 | T2 vs. T1 | 0.44 | (0.14; 1.33) | 0.151 | **0.033** |
|  |  |  |  | T3 vs. T1 | 0.24 | (0.06; 0.86) | **0.034** |  |

Model 1: MeDi score, disease duration, age, gender; Model 2: MeDi score, disease duration, age, gender, physical activity; Model 3: MeDi score, disease duration, age, gender, physical activity, income. The bold values indicate statistical significance.

Supplementary Table 6. Association between PD status and Mediterranean diet adherence after adjustment for coffee consumption.

|  | MeDi score continuous | | | MeDi score tertiles | | | | |
| --- | --- | --- | --- | --- | --- | --- | --- | --- |
|  | OR | 95% CI | p value |  | OR | 95% CI | p value | p for trend |
| Model 0 | 0.96 | (0.89; 1.04) | 0.274 | T2 vs. T1 | 0.50 | (0.19; 1.34) | 0.169 | 0.17 |
|  |  |  |  | T3 vs. T1 | 0.50 | (0.18; 1.35) | 0.173 |  |
| Model 1 | 0.95 | (0.88; 1.03) | 0.219 | T2 vs. T1 | 0.52 | (0.19; 1.38) | 0.19 | 0.139 |
|  |  |  |  | T3 vs. T1 | 0.47 | (0.16; 1.29) | 0.144 |  |
| Model 2 | 0.92 | (0.85; 1.01) | 0.072 | T2 vs. T1 | 0.56 | (0.19; 1.61) | 0.286 | 0.087 |
|  |  |  |  | T3 vs. T1 | 0.38 | (0.12; 1.15) | 0.090 |  |
| Model 3 | 0.91 | (0.83; 1.00) | 0.061 | T2 vs. T1 | 0.48 | (0.14; 1.56) | 0.228 | **0.023** |
|  |  |  |  | T3 vs. T1 | 0.21 | (0.05; 0.78) | 0.023 |  |

Model 0: MeDi score, coffee; Model 1: MeDi score, age, gender, coffee; Model 2: MeDi score, age, gender, smoking status, body mass index, physical activity, coffee; Model 3: MeDi score, age, gender, smoking status, body mass index, physical activity, education, income, coffee. The bold values indicate statistical significance.

Supplementary Table 7. Association between the non-motor symptoms and Mediterranean diet adherence.

|  | **OR per 1-point increase** | **95% CI** | **p value** |
| --- | --- | --- | --- |
| **Cognitive impairment** | | | |
| Univariate model | 0.99 | 0.91; 1.10 | 0.945 |
| Model 1 | 0.980 | 0.09; 1.08 | 0.694 |
| Model 2 | 0.97 | 0.86; 1.07 | 0.514 |
| Model 3 | 0.98 | 0.88; 1.09 | 0.730 |
| **Excessive daytime sleepiness** | | | |
| Univariate model | 0.99 | 0.89; 1.12 | 0.953 |
| Model 1 | 0.99 | 0.87; 1.12 | 0.886 |
| Model 2 | 0.99 | 0.87; 1.12 | 0.883 |
| Model 3 | 1.00 | 0.89; 1.14 | 0.062 |
| **Sleep problems** | | | |
| Univariate model | 0.96 | 0.86; 1.05 | 0.361 |
| Model 1 | 0.98 | 0.88; 1.09 | 0.658 |
| Model 2 | 0.98 | 0.88; 1.10 | 0.768 |
| Model 3 | 0.98 | 0.87; 1.09 | 0.655 |
| **Fatigue** | | | |
| Univariate model | 0.94 | 0.84; 1.04 | 0.208 |
| Model 1 | 0.92 | 0.80; 1.04 | 0.220 |
| Model 2 | 0.92 | 0.80; 1.05 | 0.220 |
| Model 3 | 0.95 | 0.84; 1.06 | 0.368 |
| **Anxiety** | | | |
| Univariate model | 0.88 | 0.78; 0.98 | **0.028** |
| Model 1 | 0.86 | 0.74; 0.97 | **0.026** |
| Model 2 | 0.86 | 0.74; 0.97 | **0.027** |
| Model 3 | 0.88 | 0.76; 0.99 | **0.043** |
| **Depressive mood** | | | |
| Univariate model | 0.93 | 0.81; 1.05 | 0.236 |
| Model 1 | 0.90 | 0.77; 1.02 | 0.116 |
| Model 2 | 0.90 | 0.77; 1.03 | 0.126 |
| Model 3 | 0.92 | 0.80; 1.05 | 0.213 |
| **Impulse control disorder** | | | |
| Univariate model | 0.99 | 0.89; 1.13 | 0.992 |
| Model 1 | 1.01 | 0.89; 1.14 | 0.901 |
| Model 2 | 0.99 | 0.88; 1.13 | 0.977 |
| Model 3 | 0.99 | 0.88; 1.12 | 0.924 |
| **Orthostatic Hypotension** | | | |
| Univariate model | 1.00 | 0.90; 1.11 | 0.998 |
| Model 1 | 0.97 | 0.86; 1.08 | 0.557 |
| Model 2 | 0.96 | 0.85; 1.08 | 0.509 |
| Model 3 | 1.00 | 0.89; 1.12 | 0.998 |
| **Urinary disfunction** | | | |
| Univariate model | 0.86 | 0.74; 0.98 | **0.029** |
| Model 1 | 0.82 | 0.69; 0.95 | **0.013** |
| Model 2 | 0.79 | 0.65; 0.93 | **0.011** |
| Model 3 | 0.83 | 0.70; 0.96 | **0.022** |
| **Constipation** | | | |
| Univariate model | 0.88 | 0.78; 0.98 | **0.027** |
| Model 1 | 0.85 | 0.74; 0.96 | **0.011** |
| Model 2 | 0.85 | 0.74; 0.96 | **0.013** |
| Model 3 | 0.88 | 0.77; 0.98 | **0.035** |
| **Pain** | | | |
| Univariate model | 0.86 | 0.74; 0.97 | **0.022** |
| Model 1 | 0.88 | 0.75; 1.01 | 0.078 |
| Model 2 | 0.89 | 0.76; 1.03 | 0.146 |
| Model 3 | 0.87 | 0.75; 1.00 | 0.066 |

Model 1: MeDi score, disease duration, age, gender; Model 2: MeDi score, disease duration, age, gender, physical activity; Model 3: MeDi score, disease duration, age, gender, physical activity, income. The bold values indicate statistical significance.
